# Supplementary material for: Improving modelling for epidemic responses: reflections from members of the UK infectious disease modelling community on their experiences during the COVID-19 pandemic
Source: Wellcome Open Res. 2024 Jan 8;9:12. [Version 1] doi: 10.12688/wellcomeopenres.19601.1 (PMC11112301; doi:10.12688/wellcomeopenres.19601.1)
Supplement: Peer review history

# Reviewer report: Reviewer response for version 1

**DOI:** 10.21956/wellcomeopenres.21712.r75342

**Published:** 2024-05-22

**Authors:** Robert Moss

**Affiliations:** 1 The University of Melbourne, Melbourne, Victoria, Australia

This article reports findings arising from a one-day workshop held in London in early 2023, in which participants were asked to reflect on the personal and professional impacts of their involvement in informing strategy and policy during the COVID-19 pandemic, for the purposes of identifying key issues (and potential recommendations to mitigate these issues) and to provide guidance for future public health crises.

 As the authors acknowledge in the introduction, there is a well-established body of literature concerning the impact of disasters on exposed persons, and ways to mitigate these impacts, but this literature primarily focuses on direct responders and survivors. Less attention has been given to persons who contribute indirectly to disaster response, such as the infectious disease modelling community represented in this article. I'm only aware of one paper that reflects on (professional) impacts of the COVID-19 response on modellers: "The COVID-19 response illustrates that traditional academic reward structures and metrics do not reflect crucial contributions to modern science", which was written in 2020 by three authors of this article.

 The common themes and personal impacts identified here may not necessarily be surprising (many reflect broader, long-standing issues in academia) but they deserve genuine attention and reflection. Many of these issues resonate strongly with my own experiences, and those of my colleagues. I wholeheartedly agree with the authors' conclusion that it is "critical we act outside of response contexts".

 The workshop was carefully planned and conducted. The supplementary materials attest to the level of engagement from the participants, and to how thoroughly the organisers have captured and reported the findings. It is unfortunate, but entirely understandable, that only a few individuals who had left the field were able to attend the event.

 I only have a few minor comments regarding the article text.

 1. In "Snapshot reflections", the third sentence begins "Affirmative responses were more common ...", referring to the question "Who should ensure that individuals are adequately supported and credited for their response work".

    My first thought was that this is not a question with a "yes" or "no" answer. It took me a moment to recall that this was a multiple-choice question, and so participants were selecting responsible organisational units from a predefined list.

    The first half of this sentence could potentially be removed, so that it begins "Respondents considered themselves (9/56), line managers (16/56), and research groups (12/56) more responsible ...".

 2. In the "Team and work dynamics" results section, the authors report that we might learn from other professions that are better adapted to emergency response work. I think this is a great suggestion, and worth highlighting as an example action for the third priority recommendation ("Ensure response teams are well-staffed, well-resourced, stable, and provided psychological support").

 3. The "Non-academic contributions" results section highlights the importance of strengthening the collaboration between academics and public health agencies. This reminds me of Pan-InfORM (Pandemic Influenza Outbreak Research Modelling), a Canadian initiative that was established in 2009 for this very purpose. The authors could cite a recent review of Pan-InfORM activities (published in 2021, doi:10.3934/publichealth.2021020) as an international example.

 4. Regarding the final sentence of the discussion:

    "It is important to discern whether these concerns are long-standing systemic issues that have been simply exacerbated by the pandemic, or if they have been particularly highlighted due to the unique stressors of the pandemic",

    I'm not sure I fully appreciate this distinction. If a concern has been "particularly highlighted" by the pandemic, I still interpret it as meaning that the concern was relevant prior to the pandemic, and I wonder if the intended meaning is that the concern was not identified or appreciated prior to the pandemic?

    This sentence also led me to expect an outline of different approaches that might be used to address long-standing systemic issues versus those were specific to the pandemic response. Otherwise I don't understand why this distinction is being made.

Is the work clearly and accurately presented and does it cite the current literature?

Yes

If applicable, is the statistical analysis and its interpretation appropriate?

Not applicable

Are all the source data underlying the results available to ensure full reproducibility?

Yes

Is the study design appropriate and is the work technically sound?

Yes

Are the conclusions drawn adequately supported by the results?

Yes

Are sufficient details of methods and analysis provided to allow replication by others?

Yes

Reviewer Expertise:

Infectious disease modelling

I confirm that I have read this submission and believe that I have an appropriate level of expertise to confirm that it is of an acceptable scientific standard.

## References

[1] : Ten years of Pan-InfORM: modelling research for public health in Canada. *AIMS Public Health* .2021;8(2) : 10.3934/publichealth.2021020 265-274 10.3934/publichealth.2021020 34017890 PMC8116193

# Reviewer report: Reviewer response for version 1

**DOI:** 10.21956/wellcomeopenres.21712.r76468

**Published:** 2024-05-03

**Authors:** Srikanth Umakanthan

**Affiliations:** 1 The University of the West Indies at Saint Augustine Faculty of Medical Sciences, Saint Augustine, Trinidad and Tobago

The authors have touched on a critical topic that was very much neglected during the COVID-19 pandemic.

 This study very well highlights the role and coordination between the employer, employee and the community using the available resources in the best possible manner.

 However the following comments need to be addressed:

 1. The keywords should not be similar to those in the title.

 2. The workshop's type, duration, and target audience should be indicated.

 3. The prior aims and objectives of the workshop and the percentage of it being achieved should be included.

 4. The professional details of the external facilitator who aided in developing the discussion topics need to be mentioned.

 5. The link between the results and the study methods needs to draw a clear conclusion. In the methods the authors look to "identify the Key issues such as lack of institutional support, insecure contracts, unequal credit and recognition, and mental health impacts" but the results look into the "to propose recommendations for future epidemic responses".  

 6. The list of specialties and the topics of the keynote speakers should be included.

Is the work clearly and accurately presented and does it cite the current literature?

Yes

If applicable, is the statistical analysis and its interpretation appropriate?

Yes

Are all the source data underlying the results available to ensure full reproducibility?

Yes

Is the study design appropriate and is the work technically sound?

Yes

Are the conclusions drawn adequately supported by the results?

Yes

Are sufficient details of methods and analysis provided to allow replication by others?

Yes

Reviewer Expertise:

Pathology, infectious diseases

I confirm that I have read this submission and believe that I have an appropriate level of expertise to confirm that it is of an acceptable scientific standard.
